# Hospitals and the War

**Published:** 1914-08-29

**Authors:** 


### THE EDITOR'S LETTER-BOX.

### Hospitals and the War.

BRIGHTON.

We are indebted to Dr. Donald Hall, assistant physician, Royal Sussex County Hospital, Major R.A.M.C.T., 2nd Eastern General Hospital, and Hon. Secretary Territorial Nursing Association, for the following statement:—

I write merely as an individual member of the staffs of the Royal Sussex County Hospital and the 2nd Eastern General Hospital in the hope of acquainting you with the facts and giving you an opportunity of putting the matter right in your next issue.

No "contingent of nurses" has been supplied by the Royal Sussex County Hospital from its present staff. The nursing staff of the 2nd Eastern General Hospital was completed in March 1909—the first Territorial hospital in the United Kingdom. All these nurses had completed their three years' general training, and vacancies have been filled as they occurred. Among these are many who have been trained at the Royal Sussex County Hospital. The present crisis has robbed us for the time being of some of our ward sisters, and these only. Fortunately, our hospital final examinations were held three months ago, and the ward sisters' places have been filled by the pick of our third-year probationers, who have just completed their training and been given their certificates. The nursing staff of the Royal Sussex County Hospital is at its full number therefore, and is not "depleted."

Miss Bird, matron of the Croydon General Hospital, was appointed principal matron of the 2nd Eastern General Hospital more than a year ago, in succession to Miss Peter, superannuated under the War Office war limit. Here, again, your information is faulty, although you guard yourself by "apparently."

At the Royal Sussex County Hospital board meeting of August 5 it was decided to offer the resources of the hospital to the Government for the use of our wounded and sick soldiers and sailors to the limit consistent with the needs of Brighton and the county. It is surely obvious that many of the chronic surgical cases and some medical can easily have their admission postponed. Extra beds and equipment were obtained, and an offer of 100 beds has been made to the War Office. No ward has been closed; they are all fully staffed and in full working order, but the number of in-patients has been diminished from 200 to 140 (approximate figures), this solely to help if called upon in this national crisis, and not for the purpose of hoarding the hospital's funds. We had a legacy of £10,000 free of duty only last month.

As regards the Royal Sussex County Hospital staff, the senior physician, pathologist, and secretary are all out of Brighton with Territorial regiments. One physician, two surgeons, two assistant physicians, two assistant surgeons, and the senior anæsthetist are on duty at the 2nd Eastern General Hospital and doing their work at the Royal Sussex County Hospital as well. Of these I am one. I myself was in charge of two-thirds of the medical beds, and discharged the patients who were fit to leave on August 6. Not one but left willingly and cheerfully, knowing the reason wherefore, and with every case arrangement was made for continuation of the treatment.

Where the lack of "administrative perception and energy" comes in, I fail to see. At Brighton we have done our best, and I hope you will now see your way to say so.

*** [Dr. Donald Hall and all who have worked for the 2nd Eastern General Hospital at Hove have done excellent work, and done it, too, with commendable expedition. Our remarks in The Hospital, August 15, p. 548, did not refer to this Territorial hospital's work or directly to anyone connected with it. They were directed mainly to the temporary closing of certain wards in the Royal Sussex County Hospital, as publicly certified by its Chairman. The Chairman distinctly stated in his letter to the papers that the call upon the contingent of nurses for military service had been made.—Ed. The Hospital.]

### The War and University Studies.

*To the Editor of* The Hospital.

